# The benefits of nurturing care interventions on early child development and care: findings from a quasi-experimental study in a humanitarian setting

**DOI:** 10.1186/s12887-023-04239-z

**Published:** 2023-08-24

**Authors:** Viktoria Sargsyan, Ana Tenorio, Mediatrice Uwera, Andre Gasirikare, Jean Aime Habyarimana, Jennifer S Salcido, Christy Felner, Muneera A. Rasheed

**Affiliations:** 1 World Vision International, Global Educator Sector Team, Yerevan, Armenia; 2 World Vision International, Rwanda Office, Kigali, Rwanda; 3 World Vision International, US Office, Washington, DC USA; 4 Department of Global Public Health and Primary Care, University of Bergen, Bergen, Norway

## Abstract

**Background:**

The study objective was to determine if a nurturing care parenting intervention delivered in a humanitarian setting in Rwanda would benefit early development, learning, and care outcomes for young children under five years and their caregivers compared to standard care.

**Methodology:**

Rwanda’s Mugombwa, Kansi, and Kigeme refugee camps and host communities implemented the parenting program. Via a quasi-experimental research design, the study assessed the effects of intervention delivered as a high dose (HD: 12 group sessions and four home visits) or low dose (LD: 6 group sessions and two home visits) on child and caregiver outcomes compared to the control group from similar settings receiving standard care. The Ages and Stages Questionnaires-3 (ASQ-3) assessed child development outcomes. The Multiple Indicator Cluster Survey questionnaire assessed parenting practices concerning early learning and stimulation.

**Findings:**

The assessment included 733 children and families in total: HD = 314, LD = 240, control = 179. The researchers found no significant difference in child development scores between the intervention and control groups. Significantly higher proportion of caregivers exposed to HD and LD packages had engaged in early learning and stimulation practices compared to the control group, respectively, with 211(67.2%), 148 (61.7%) vs. 66 (36.9%), p < 0.001 caregivers engaged in 4 or more activities in the past three days. Similarly, on responsive feeding items, a higher percentage of HD and LD group caregivers were engaged in positive behaviours compared to the control group: 164 (52.2%), 108 (45%) vs. 62 (34.6%), p = 0.001. The study found no difference between the study arms regarding caregiver mental health.

**Conclusion:**

Parenting programmes in humanitarian settings can improve nurturing care practices, even with a low dose, which is essential to strengthening children’s resilience in at-risk conditions. Further studies in humanitarian contexts are crucial to understand the implementation needs in sensitive contexts.

**Supplementary Information:**

The online version contains supplementary material available at 10.1186/s12887-023-04239-z.

## Introduction

The first five years of life are the most important for long-term learning, health, and development, as brain growth peaks during this time [1]. Both negative and positive experiences in the early years affect the brain pathways for cognition, self-esteem, emotional regulation, impulse control, and planning abilities [2]. Early childhood is foundational for numerous global health and social issues, encompassing mental illness, stunting/obesity, heart disease, criminality, and substance abuse [3, 4]. Early life experiences, such as responsive interaction with parents/caregivers, health and nutrition, and a loving, stable, low- stress and safe environment, play a critical role in determining the capacity of a baby’s brain development and learning. Such positive experiences lay a foundation for a baby’s future as a healthy, productive, well-rounded adult. As the baby’s first teachers, first nurturers, and first protectors, the role of the baby’s primary caregivers in creating a positive environment for the baby’s growth and development is crucial [2, 5]. While nutrition and health care are vital, children also need a caring, responsive, and protective environment to ensure they develop to their full potential. Building parents’ and caregivers’ confidence and capacity to raise happy, healthy children is the key to holistic child development [6]. The Nurturing Care Framework (NCF), launched in 2018, a roadmap to promote holistic child development outcomes also recognized these intervention components, i.e., early learning, responsive caregiving, safety and security, along with health, and nutrition [7].

An entire generation’s potential risks can arise from adverse experiences during early childhood, including forced migration and emergencies resulting from disasters, war, or conflict [8]. Globally, the number of children living in conflicts and war zones is increasing. As of mid-2022, there are around 103 million forcibly displaced people worldwide, of which 32.5 million are refugees, 53.2 million are internally displaced, 4.9 million are asylum seekers, and children make up almost 50% of the total affected population [9]. According to UNICEF, 29 million babies were born in crisis settings in 2018 [10]. In emergency response, early childhood development (ECD) is one of the least prioritized area by humanitarian agencies. Allocated funding for Early Childhood Development (ECD) programs targeting children under the age of five is minimal or nonexistent. A report indicated that ECD was not considered in 60% of active humanitarian responses [11].

Early childhood interventions informed by NCF can serve as a buffer against the risks for poor development in humanitarian contexts where children experience multiple risk factors [12]. Parents and primary caregivers have been recognized with a central role in making NCF operational by providing nurturing care parenting practices to promote young children’s brain development. These practices also provide protection from harmful effects of adversities children might face in such situations [13, 14]. While ECD-focused parenting interventions to strengthen the resilience of families and children in humanitarian response settings are of critical importance, the interest of the donors and hence, investment in such interventions needs to be improved. ECD funding represented only 3.3% of total aid development that went to crisis-affected countries in 2017 [15] and the figures have remained the same for 2020 [16]. The potential obstacles could be limited evidence about the effective ECD programs, their parameters such as implementation data describing the critical process and factors essential for delivering high-impact interventions, and effective measurement tools, which could guide the set-up and roll-out of such interventions in humanitarian contexts [12, 17].

World Vision in Rwanda (WVR) implemented a parenting project called Care and Comfort for Children (3 C) to strengthen the resilience of families and communities to improve early childhood development outcomes of under-five age children living in refugee settings. The project served as an opportunity to fill the evidence gap by answering some questions about the optimum dosage that could inform the global community of practice regarding the critical components of the ECD parenting programme in refugee settings. The study’s primary objective was to examine the effect of the 3 C parenting program on under-five children and their caregivers in a humanitarian setting in Rwanda. The research questions were primarily focused on assessing the difference between two intervention groups, high dose (HD) and low dose (LD) receiving groups, compared to the standard care in a humanitarian context, and answering the following questions:

- Will children of caregivers who receive intervention demonstrate improved developmental outcomes compared to children who receive a standard service? Will the three study arms be significantly different in child development outcomes?
- Will caregivers who receive intervention demonstrate improved caregiving practices for optimal ECD compared to caregivers who receive standard service as indexed by differences in (i) engagement with play activities (ii) quality of learning environment at home (iii) responsive feeding practices? Will there be a difference between the HD and LD intervention groups in caregiving practices?
- Will caregivers exposed to the intervention (HD or LD) have reduced symptoms of anxiety and depression compared to caregivers who receive standard services? Will there be a difference between the two intervention groups?

## Methods

### Study participants

**Fig. 1:**
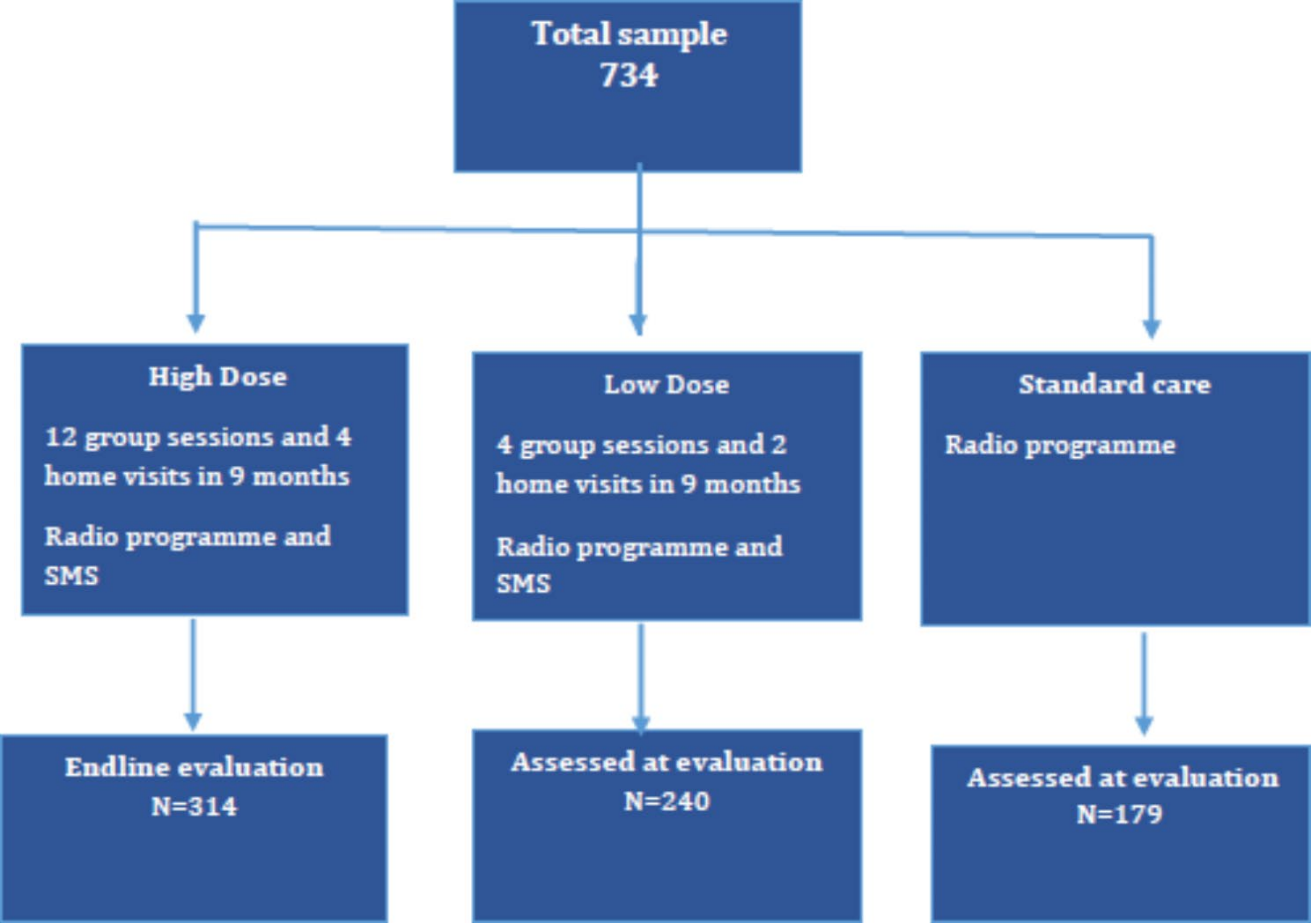
Intervention and control groups of the 3 C study

The 3 C project targeted refugees settled in the Mugombwa refugee camp in Gisagara District, established in 2014, and three neighboring host sectors - Kansi, Kigeme, and Mugombwa. Mugombwa refugee camp has a population of 10,574, consisting of 2,260 households which have 2,014 under-five age children. The host communities of Kansi, Kigeme, and Mugombwa have a total population of 61,399, with around 17% of the population being children under-five age (10,203). The project team intended to reach 1,500 households with 3,000 children under five (CU5). The families were primarily selected based on their needs and interest in participating in the 3 C project and as per the set criteria. The targeted households had to meet the following eligibility criteria: having at least two children under-five age; being interested in participating in the project; belonging to the lowest wealth category, as defined by the Government for the Rwandan population. For the control group, the research consultancy team with World Vision selected a site based on feasibility of operations with similar demographic characteristics [Fig. 1].

### Research design and sampling framework

The researchers designed the evaluation as a quasi-experimental non-equivalent control group post-test-only research [18, 19]. The research aimed to assess the effect of ECD parenting programme for children under five, particularly measuring the effect of HD and LD parenting packages on child and caregiver outcomes in refugee settings (host and camp). The intervention section includes details of the intervention and dosage.

The researchers collected data in three study arms selected from host and camp communities. A sample size of 176 per group was identified at a power of 0.80 and level of significance of 0.05 to detect a difference in 15 points between low dose group and control on a standard child development test (the Ages and Stages Questionnaire-3) based on a previous trial using ASQ 3 in India [20]. The sample size was calculated using Stata v16. World Vision provided the list of households with children aged 0–5 years living in targeted intervention sites and control sites, and the research consultancy team selected the study respondents using a random sampling approach. The Institutional Review Board of the College of Medicine and Health Sciences, University of Rwanda, approved the research study (Approval No. 180/CMHS IRB/2022). The head of the household (legal guardian of the participants) provided informed consent for participation in the study. The study team conducted research activities following the institutional guidelines.

### Intervention

The main objectives of the 3 C intervention were to strengthen: (1) Family & Caregiving Environments for Children 0–5; and (2) Community Environment for Children 0–5. Go Baby Go (GBG) parenting program developed by World Vision International [21] served as a foundation for the 3 C project. The GBG model strengthens knowledge, skills, and resilience-promoting parenting behaviours to provide nurturing care during the first 1,000 + days of life. The GBG is aligned with the Nurturing Care Framework and primarily focuses on early learning, responsive caregiving, and safety and security of nurturing care domains. The 3 C also included Sesame Workshop ECD materials for 4–6 age groups, including videos from Sesame Workshop’s “I Elmo” and “WASH UP!“ series and accompanying in-class and take-home activities.

The HD group received 12 caregiver group sessions and four home visits, while the LD group received four caregiver group sessions, and two home visits. The themes covered in HD and LD in the different intensity were: sensitive & responsive caregiving; nurturing a holistic child development; play-based parenting; WASH, child growth (protection for infections, responsive feeding); positive discipline, creating safe home spaces, and protecting from violence; responding to children in crisis via responsive and loving care; father’s engagement; COVID 19; caregiver self-care.

Both groups received exposure to 15 radio sessions and 12 ECD SMS messages. Camp leaders and local leaders recruited 44 community facilitators who were refugees. The trained and supported community facilitators delivered the 3 C: 32 facilitators delivered HD and 12 delivered LD packages, and got support from nine Mentors (six for HD and three for LD). The two Project Staff (one for host communities one for camp) roles were to train, monitor and supervise 3 C implementation in host and camp settings for fidelity and quality, provide the workforce with supportive supervision, and address the emerging challenges.

The 3 C project started in September 2020 and finished in January 2022. The following were the stages of the study:

- Preparations, which consumed six months for the ECD curriculum’s adaptation, and translation (1.5 months); defining the high dose and low dose packages; Sesame Workshop (SW) material adaptation and translation (6 months); training of the workforce in 3 C and SW integrated curriculums for group sessions and 3 C home visiting curriculum; developing research and baseline protocol.
- The implementation stage lasted seven months, from August 2021 until January 2022. Overall, 125 caregiver groups (12 caregivers per group) received the two packages: 83 HD and 42 LD groups in host and camp communities.
- The evaluation started in March 2022, with one week of training and data collection, and the trained enumerators collected the field data in two weeks.

### Data collection measures and procedures

Different tools served to measure child and caregiver outcomes. The research team translated the data collection forms into Kinyarwanda. The study used the following tools, explained below.

Child development: Ages and Stages Questionnaire-3rd edition [22] to measure child development outcomes. The ASQ-3 comprises five domains of development: gross motor skills, fine motor skills, communication skills, problem-solving skills, and personal-social development. Each domain includes six items. Different questionnaires are available for different age intervals. The ASQ has been used previously in Rwanda to assess the developmental outcomes of preterm infants [23]. The publisher approved the translated and back-translated forms and granted permission to use the forms.

In the current study, three scales of the ASQ-3 (gross motor, fine motor, and problem-solving) were administered as direct child performance assessment in the presence of the caregivers. The ASQ-3 is scored by assigning points to each of the 30 items on the age-specific ASQ-3 form. An item is scored as zero points if the child is not yet able to perform the task/behavior, five points if the child sometimes can, and ten points if the child is consistently able to perform the task/behavior. The points are then added up for a maximum possible score of 300 on the ASQ-3.

A quick psychometric analysis was conducted prior to outcome analysis. Reliability analysis of ASQ 3 indicated a Cronbach alpha of 0.93 for the total score. For the individual domains, the reliability scores ranged from 0.67 for personal-social to 0.81 for the communication score.

Caregiver nurturing practices: Parent engagement with early learning and stimulation included items from the UNICEF Multiple Indicator Cluster Survey (MICS) [24]. It includes six different activities scored as yes or no (In the past three days, did you or any household member over 15 years of age engage in any of the following activities with your child: read books or looked at pictures together, told stories, sang songs, took the child outside of the home compound, play with the child, named or counted or drew things to or with child). A response of yes to four or more activities was used as an indicator of engaged caregivers. Cronbach alpha was 0.81, 0.88, and 0.91 for maternal, paternal, and other caregiver engagement items, respectively. A commonly used questionnaire in LMICs for responsive feeding practices was administered. It included eight different behaviors, e.g., the caregiver encourages the child to eat during the meal, or the caregiver asks about the food, and reported as rarely, sometimes, or always. Data were analyzed as a percent of families reporting per group. Cronbach alpha for these items was 0.84.

The learning environment at home: Selected items from the Home Observation for Measurement of Environment Inventory, Infant Toddler version (HOME-IT) [25] was used to measure the quality of the home environment across different domains: access to play materials, opportunities for outings, father involvement and participation in ECD center. Since the whole scale was not used, analyses were completed as numbers and percentages per item.

Caregiver mental health: The Hopkins Symptoms Checklist (HSCL)-25 [26], a widely used screening tool, was used to measure caregiver mental health. The HSCL-25 measures symptoms of anxiety and depression. It consists of 25 items: Part I of the HSCL-25 has ten items for anxiety symptoms; Part II has 15 for depression symptoms. The scale for each question includes four categories of response (“Not at all,“ “A little,“ “Quite a bit,“ and “Extremely,“ rated 1 to 4, respectively). Two scores were calculated: the total score is the average of all 25 items, while the depression score is the average of the 15 depression items. A mean score of 1.75 (sum score divided by the number of items) was defined as the cut-off point for syndromal depression and anxiety based on previous studies [27, 28]. Inter-rate reliability was high, with Cronbach’s alpha of 0.92 for the tool.

Data on socioeconomic status and other demographic variables such as maternal and paternal education, employment status, and identification of primary caregivers were also collected.

### Data collection procedures

The study team collected the evaluation data during the period of 14–25 March 2022. An external consultant trained the enumerators (a total of 17) in five days from 7 to 11 March 2022. The training components entailed core concepts of ECD, data collection and management protocols, and two-day field practice. During data collection, each enumerator assessed at least four caregivers per day; the average length of interview per caregiver was 1-1.5 h. The research training team, the monitoring and evaluation officers, and the project officer supervised and provided enumerators with technical and operational support. Identifying families was supported by ‘community guides’ who were part of the intervention delivery teams. The head of the household (legal guardian of the participants) provided informed consent before data collection.

Data were collected digitally using tablets with the questionnaires pre-programmed in KOBO Collect. During interviews, the data manager regularly checked the consistency of data collected by enumerators to mitigate potential mistakes, confirm that the survey protocol was followed, and provide comments to the field officers for correction and improvement. At the end of each workday, all enumerators submit the collected data to the server for checking. The data manager, first of all, confirmed that the coding of all sheets was correct. Following this cross-checking, the data manager provided daily feedback to correct mistakes made by any enumerator.

### Data analysis

Analysis was completed by an independent researcher using the Stata v17. The significance level was taken as p = < 0.05. Mean and standard deviation (SD) were computed for continuous variables and proportions for categorical. ASQ 3 was used as a continuous score. In addition to the total score, domain-specific scores were calculated (communication, fine and gross motor, problem-solving, and personal-social). Generalized Linear Modeling (GLM) for ANOVA assessed the group differences on continuous variables and GLM binomial for categorical variables. Analysis was adjusted for SES, caregiver education, child sex and preschool attendance.

### Findings

Data was collected from a total of 733 families, with 314 in the HD group, 240 in the LD, and 179 in the control group. Table 1 below shows the socio-demographic characteristics of the research participants collected from the three study arms (control, high/low dose). No significant differences were observed between the study groups in terms of the number of children within the household or the educational background of the caregivers. Child characteristics, i.e., age and sex, were also non-significant. However, SES differed between the three groups, with a considerably lower ‘poorest’ category in the control group (6.9%) compared to 23.3% in HD and 26.7% in LD. The LD group had the highest percentage (28.3%) of the least poor category.

**Table 1:** Socio-demographic characteristics by study arms

	Control	High dose	Low dose	p-value
	N = 179	N = 314	N = 240	
No. of people the household	6.4 ± 2.3	6.0 ± 2.1	6.4 ± 2.2	0.18
No. of children ≤ 17 years old living in the household	3.5 ± 1.6	3.6 ± 1.7	3.7 ± 1.6	0.49
If mother has ever attended school				
Yes	120 (69.0)	238 (75.8)	168 (70.0)	0.17
No	54 (31.0)	76 (24.2)	72 (30.0)	
Wealth Index (tertiles)				
Very poor	41 (23.6)	119 (37.9)	94 (39.2)	< 0.001
Poor	84 (48.3)	96 (30.6)	55 (22.9)	
Least poor	49 (28.2)	99 (31.5)	91 (37.9)	
Child sex				
Male	90 (50.3)	160 (51.0)	118 (49.2)	0.92
Female	89 (49.7)	154 (49.0)	122 (50.8)	
Child age				
8–24 months	79 (44.1)	107 (34.1)	80 (33.3)	0.096
25–48 months	72 (40.2)	137 (43.6)	103 (42.9)	
49–66 months	28 (15.6)	70 (22.3)	57 (23.8)	

Note: Data is presented as Mean (SD) or N (%) as appropriate

The results below answer the study proposed research questions. Outcome analysis indicated no significant differences between intervention and control groups in total ASQ 3 scores and between the HD and LD receiving groups [Table 2].

**Table 2:** Child development scores on ASQ-3 by study arms

	Control N = 179	High dose N = 314	Low dose N = 240	Total N = 733	p-value
Communication	38.3(17.7)	37.3 (14.2)	36.4 (18.1)	36.6 (18.6)	0.315
Gross motor	42.4 (14.3)	41.5 (11.4)	40.7 (14.6)	42.5 (15.0)	0.276
Fine motor	42.2 (15.1)	42.6 (12.1)	42.9 (15.5)	42.8 (15.9)	0.633
Problem solving	33.5 (11.9)	33.0 (9.6)	32.5 (12.3)	33.6 (12.6)	0.387
Personal social	40.8 (14.8)	40.8 (11.8)	40.9 (15.2)	42.1 (15.6)	0.912
Total	202.9 (50.8)	201.1 (40.6)	199.2 (52.0)	203.5 (53.4)	0.498

Note: Data is presented as Mean (SD), and adjusted for SES, maternal education, child sex and preschool attendance

Examination of the ASQ scores with age revealed an increased score for the older age groups in the intervention groups compared to the control [Table 3]. However, the differences were not significant. The only significant positive trend was identified in the personal social domain among 25–48 month old children in favor of the intervention group.

**Table 3:** ASQ 3 scores by child age groups

8–24 months	Control N = 78	High dose N = 105	Low dose N = 80	p-value
Communication	31.4 (18.3)	28.7 (17.02)	25.9 (16.9)	0.142
Gross motor	35.7 (17.3)	35.1 (17.71)	30.6 (18.6)	0.101
Fine motor	40.1 (17.2)	40.8 (19)	38.1 (16.4)	0.503
Problem solving	31.5 (13.9)	31.5 (14.08)	27.6 (13.6)	0.114
Personal social	41.8 (17.9)	42.7 (17.5)	35.9 (18.5)	0.029
Total	187.0 (64.6)	185.4 (62.31)	164.1 (54.9)	0.026
25–48 months	Control N = 73	High dose N = 139	Low dose N = 102	p-value
Communication	38.1 (21.6)	38.2 (23)	40.1 (21.2)	0.776
Gross motor	49.4 (14.1)	50.7(12.7)	51.4 (9.5)	0.572
Fine motor	40.5 (18.8)	43.5 (15.3)	44.2 (14.4)	0.29
Problem solving	33.8 (14.2)	36.5 (13.8)	36.6 (13.3)	0.347
Personal social	41.2 (14.5)	45.9 (13.6)	46.7 (12.2)	**0.017**
Total	208.5 (59.5)	220.1 (58.5)	224.7 (44.9)	0.149
49–66 months	Control N = 28	High dose N = 70	Low dose N = 57	p-value
Communication	45.7 (11.4)	45.8 (12.4)	45.4 (11.5)	0.978
Gross motor	40.5 (15.2)	41.0 (15.4)	40.4 (15.2)	0.976
Fine motor	44.8 (12.6)	47.1 (13.5)	47.7 (13.2)	0.629
Problem solving	33.2 (8.7)	34.5 (8.3)	33.9 (7.4)	0.76
Personal social	35.5 (15.6)	38.4 (14.9)	40.0 (14.6)	0.432
Total	203.75 (33.46)	212.57 (41.93)	213.77 (37.85)	0.511

Note: Data presented as Mean (SD)

For further test of validity, we examined gender differences and maternal education, and preschool attendance. No differences were found between boys and girls except for the communication domain, with girls (Mean 38.6) scoring slightly higher than boys (Mean 34.7) (Supplementary Table S1). When examining caregiver education and preschool attendance, those indicated significant differences in the right direction (Supplementary Tables: S2, S3). Children of educated mothers scored 13 points higher than those of uneducated mothers, with a mean of 207 (SD 56.7) vs. mean 194.8 56.1, respectively, p = 0.009. In contrast, children attending preschool had a 34 points higher score on total ASQ 3 than those who were not, with a mean of 224.4 (SD 42.8) vs. 188.3 (SD 60.7), p = 0.000, respectively.

**Fig. 2:**
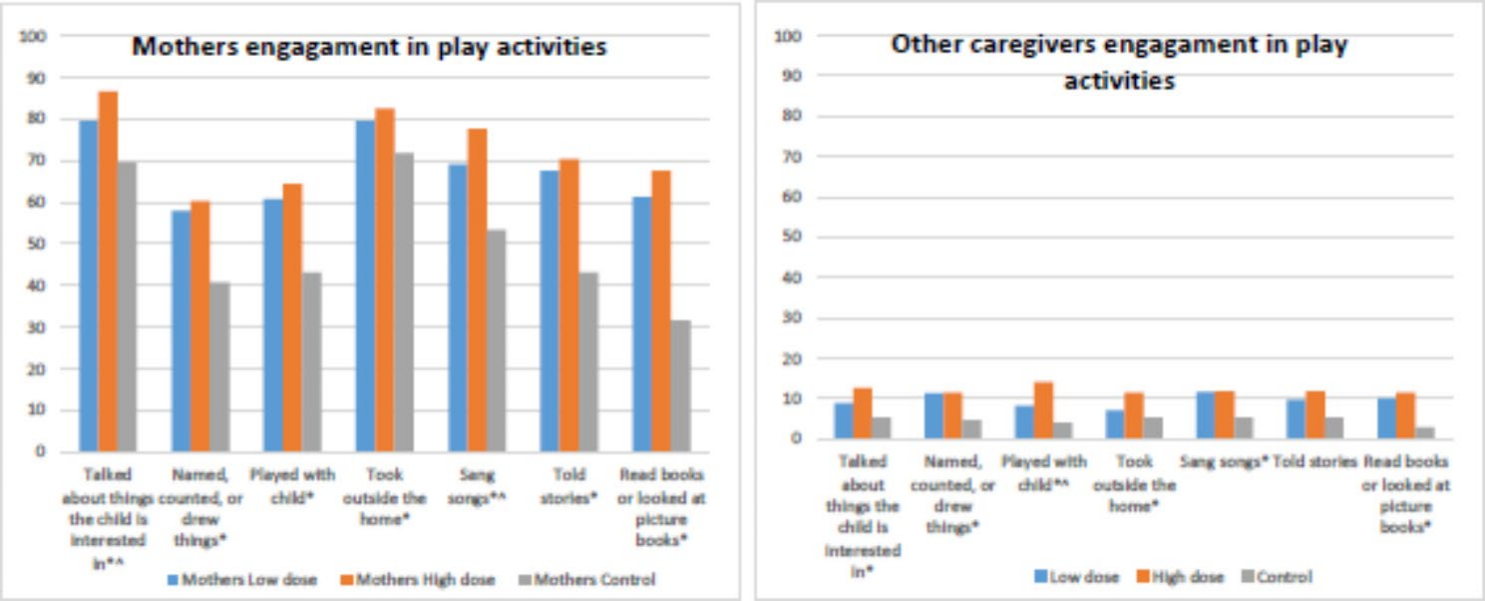
Maternal and other caregivers’ engagement with play activities in the past 3 days by study arms Note: Data is presented as %. All items were significant at p < 0.05 denoted as * for three groups, and ^ for high dose vs. low dose. Analysis was adjusted for caregiver education, wealth index, child age and child sex

Significantly higher proportion of mothers exposed to the HD and LD engaged in early learning and stimulation practices compared to the control group respectively, with 211(67.2%), 148 (61.7%) vs. 66 (36.9%), p < 0.001 mothers engaged in 4 or more activities in the past three days. The findings were significant for all play and stimulation items [Fig. 2]. Significant differences were found between HD and LD groups only for two activities: sang songs (77.7% vs. 69.2%, p = 0.024) and talked about things that interested the child (86.6% vs. 79.6%, p = 0.028). For the Other caregivers in the house, significant differences were found for all play and stimulation activities except one item, with a more significant number of other caregivers in the intervention groups reporting engagement in play and stimulation activities compared to the control [Fig. 2].

No difference was found regarding the father’s engagement with play activities between the groups in any of the seven items. Also, the analysis by child sex and age indicated similar effects.

**Fig. 3:**
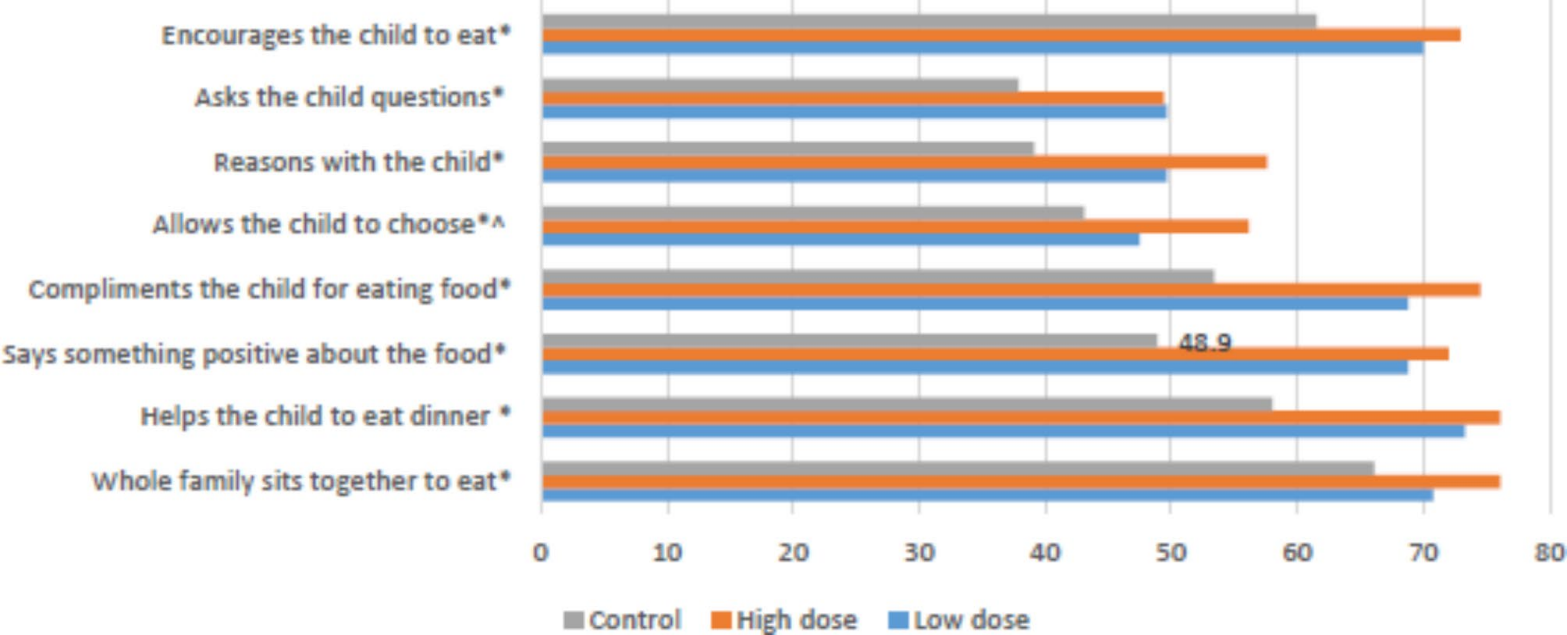
Comparison of intervention and control groups on responsive feeding behaviors Note: Data is presented as % for ‘most of the time’. All items were significant at p < 0.05 denoted as * for three groups and ^ for high dose vs. low dose. Analysis was adjusted for caregiver education, wealth index, child age and child sex

Regarding responsive caregiving, the results show a significantly greater number of caregivers in the intervention groups, both HD and LD, reporting positive behaviors on all eight items compared to the control group [Fig. 3]. Examination by child sex and age revealed similar effects for both.

Concerning playing with toys, a greater number of children in both intervention groups had homemade toys compared to children in the control group (60.3% vs. 90.1% in HD and 88.3% in LD, p = 0.000) and also toys from a shop (22.4 vs. 80.3% for HD and 79.2 in LD, p = 0.000) [Table 4]. Attendance in ECD centers (for children between 2 and 4 years) was significantly different for the intervention group compared to the control (28.2% vs. 42.3% in HD, 53.3% in LD, p = 0.000). However, ECD center attendance was the same for the older age group and between boys and girls.

**Table 4:** Items related to learning environment at home by intervention and control

	Study arms			
	Control N = 174	High dose N = 314	Low dose N = 240	p-value
The child plays with Homemade toys Toys from shop Household items	105 (60.3) 39 (22.4) 154 (88.5)	283 (90.1) 252 (80.3) 287 (91.5)	212 (88.3) 190 (79.2) 218 (90.8)	0.000 0.000 0.128
No. of picture books for the child (Mean, SD)	0.17 (0.4)	4.3 (0.22)	0.77 (0.12)	0.000
Time spent with child during play in a day None About 15 min About 30 min About 60 min More than 60 min	14 (8.1) 47 (27.0) 44 (25.3) 32 (18.4) 27 (15.5)	1 (0.32) 69 (21.9) 95 (30.3) 60 (19.1) 85 (27.0)	5 (2.1) 60 (25.0) 81 (33.8) 33 (13.8) 50 (20.8)	0.000
Talks with child when busy Showed/taught child something new past week Found something new for child to play with	104 (59.7) 69 (39.6) 23 (13.2)	218 (69.4) 218 (69.4) 164 (52.3)	174 (72.5) 171 (71.3) 104 (43.3)	0.019 0.000 0.000
Attendance in ECD program (all) Age group 25–48 months Age group 49–66 months	49 (28.2) 25 (34.3) 22 (81.5)	133 (42.3) 62 (44.6) 64 (91.4)	128 (53.3) 64 (62.8) 54 (94.7)	0.000 0.000 0.140

Note: Data is presented as N (%) unless stated and is adjusted for SES, maternal education, child sex and preschool attendance

Analysis of caregiver mental health data, measuring at-risk for depression and anxiety, indicated no significant differences between the three study arms, with 5.6% in control, 3.5% in HD, and 3.3% in LD found at-risk of depression (p = 0.43), and with 5.59% in control, 4.14% in HD and 3.75% in LD for experiencing anxiety (p > 0.5).

## Discussion

The study aimed to determine if the NCF-aligned parenting program implemented in a humanitarian context can benefit ECD outcomes for children and their caregivers. The research questions explored the benefits of 3 C delivered in high and low-dose packages compared with the control group with similar socio-demographic characteristics receiving standard services. Findings indicated no significant differences between the intervention and control groups on the main outcome measure, i.e., ASQ 3. The fact that no differences were identified in the outcomes could be attributed to different implementation factors, such as the length and quality of the intervention. A previous evaluation of the Go Baby Go parenting project in Armenia found benefits for child development (a non-humanitarian context) [29]. The study duration was similar to the 3 C (8 months of implementation, with a maximum of six direct interactions with the targeted beneficiaries). However, the context was different, and there could possibly be a difference in the quality of implementation.

Moreover, in the Armenia study, the outcome measurement tool used for the final evaluation (Bayley Scales of Infant Development-III) could be more sensitive than ASQ-3. A parenting intervention trial from the Rwandan context [30] reported differences using ASQ 3 when used as a parent report measure but did not find benefits with a direct assessment measure (Malawi Development Assessment Tool). Similarly, the 3 C study used direct child performance, which could increase tool sensitivity and reduce caregiver report bias. Some studies report poor agreement between parent and assessor scores on ASQ 3 [31].

There were statistically significant differences between the two interventions and the control groups on maternal practices for early learning and responsive caregiving and feeding, with both showing a significantly higher percentage of caregivers reporting positive practices. This result indicates that even the LD intervention, with fewer direct interactions with caregivers and delivered at a lower frequency (one group meeting per 1.5-2 months), could change nurturing care parenting practices. No differences were found in caregiver mental health between the intervention and control groups. The no change could be due to multiple support interventions and services available for caregivers in both intervention and control settings.

The evidence of differences in practice suggests a positive change in general parenting and interaction with children. Since the change in practice is the main pathway to ECD outcomes, we can assume that given greater intensity or length of intervention, those could have translated to outcomes for children. The positive change in family practices aligns with the overwhelming evidence on parenting interventions in community-based settings [5]. Concerning evidence from humanitarian settings, a pilot randomized controlled trial was conducted in refugee camps in Lebanon aimed to evaluate the benefits of a parenting education programme delivered through group sessions. The authors found positive changes in maternal disciplinary practices [32].

There were several limitations of the study and pertaining to the research design. The quasi-experimental non-equivalent group control group post-test only research design was used due to feasibility, ethical considerations, and the possibility of high mobility in a humanitarian context. The major limitation is the lack of baseline data which threatens the study’s internal validity. Due to the lack of information on baseline characteristics, it is difficult to ascertain whether the observed changes from baseline to end-line were solely attributed to the intervention. Additionally, the differences in caregiver characteristics at the end-line could be influenced by the fact that caregivers may have started at different points in the various groups, which could contribute to the observed variations. Another limitation was that the outcome measure (ASQ 3) was not validated for the setting. Nonetheless, the findings are valuable for the field of ECD interventions, specifically the benefits of the programme with different dosages. Parenting programmes in humanitarian settings delivered by staff trained in the community can have a positive effect. An RCT with two intervention arms (HD and LD) is recommended for a more robust evaluation. A comprehensive implementation evaluation needs to be integrated to be able to answer burning questions for ECD in humanitarian settings.

### Electronic supplementary material

Below is the link to the electronic supplementary material.

Supplementary Material 1: Additional analysis for Ages and Stages Questionnaire-3

## Data Availability

The datasets generated and/or analysed during the current study are not publicly available given the sensitive nature of information but are available from the corresponding author on reasonable request.
