# Supplementary material for: The benefits of nurturing care interventions on early child development and care: findings from a quasi-experimental study in a humanitarian setting
Source: BMC Pediatr. 2023 Aug 24;23:419. doi: 10.1186/s12887-023-04239-z (PMC10463768; doi:10.1186/s12887-023-04239-z)
Supplement: Supplementary file 1 — Supplementary Material 1: Additional analysis for Ages and Stages Questionnaire-3 [file 12887_2023_4239_MOESM1_ESM.docx]

**Supplementary File**

Table S1: ASQ 3 scores (Mean, SD) by child gender

|  | Boys  N=368 | Girls  N=361 | p-value |
| --- | --- | --- | --- |
| Communication | 34.7 (19.9) | 38.6 (19.2) | **0.006** |
| Gross motor | 42.3 (16.9) | 42.7 (16.4) | 0.796 |
| Fine motor | 42.7 (16.1) | 43.2 (16.3) | 0.774 |
| Problem solving | 32.7 (12.8) | 34.5 (13.0) | 0.073 |
| Personal social | 41.7 (16.1) | 42.5 (15.5) | 0.512 |
| Total | 200.0 (57.3) | 207.3 (56.1) | 0.085 |

**Table S2: ASQ 3 scores in the total study population by caregiver education (ever attended school)**

|  | No  N=202 | Yes  N=527 | p-value |
| --- | --- | --- | --- |
| Communication | 35.3 (19.32) | 37.2 (19.8) | 0.246 |
| Gross motor | 41.1 (16.6) | 43.0 (16.73) | 0.172 |
| Fine motor | 40.4 (16.3) | 43.8 (16.03) | **0.012** |
| Problem solving | 31.83 (13.2) | 34.3 (12.7) | **0.021** |
| Personal social | 40.4 (15.9) | 42.7 (15.7) | 0.072 |
| Total | 194.8 (56.1) | 207.0 (56.7) | **0.009** |

Note: Data is presented as Mean (SD)

**Table S3: ASQ 3 scores in the total study population by preschool attendance**

|  | No  N=163 | Yes  N=151 | p-value |
| --- | --- | --- | --- |
| Communication | 29.4 (22.2) | 48.9 (16.7) | **0.000** |
| Gross motor | 47.3 (13.5) | 54.3 (9.1) | **0.000** |
| Fine motor | 42.5 (17.2) | 43.8 (14.4) | **0.024** |
| Problem solving | 34.6 (14.3) | 37.5 (12.6) | 0.121 |
| Personal social | 43.9 (14.3) | 46.6 (12.5) | 0.086 |
| Total | 203.1 (59.4) | 237.1 (42.7) | **0.000** |

Note: Data is presented as Mean (SD) for children older than 2 years
